# Supplementary material for: Saturated Transposon Analysis in Yeast as a one-step method to quantify the fitness effects of gene disruptions on a genome-wide scale
Source: PLoS One. 2025 Feb 6;20(2):e0312437. doi: 10.1371/journal.pone.0312437 (PMC11801604; doi:10.1371/journal.pone.0312437)
Supplement: S1 File — (PDF) [file pone.0312437.s001.pdf]

References

1. Laan L, Koschwanez JH, Murray AW. Evolutionary adaptation after crippling cell polarization follows reproducible trajectories. eLife. 2015;4. doi:10.7554/elife.09638.
