# Supplementary figures and images for: Saturated Transposon Analysis in Yeast as a one-step method to quantify the fitness effects of gene disruptions on a genome-wide scale
Source: PLoS One. 2025 Feb 6;20(2):e0312437. doi: 10.1371/journal.pone.0312437 (PMC11801604; doi:10.1371/journal.pone.0312437)

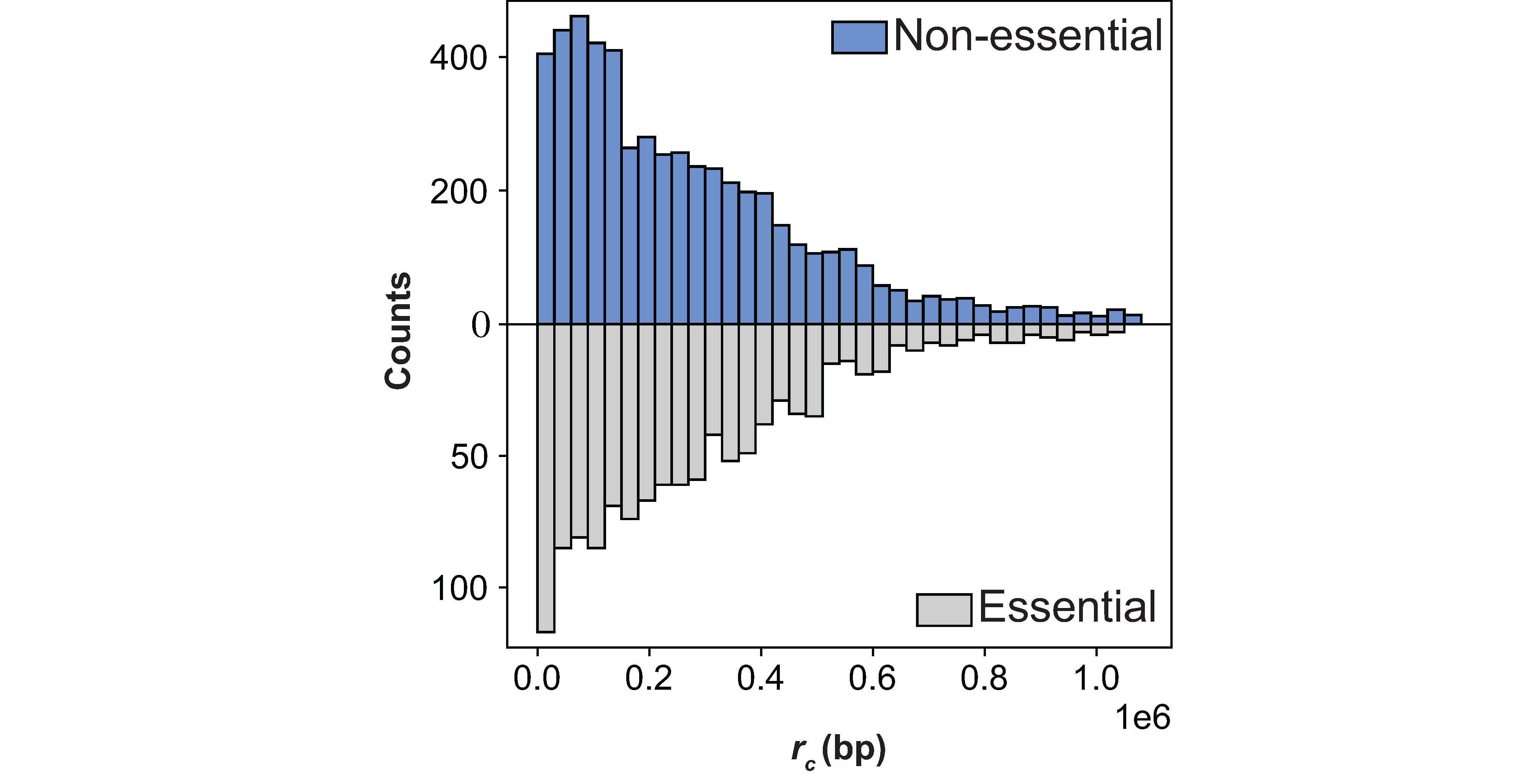

Supplement: S1 Fig — Plot of the number of essential and non-essential genes for a specified distance rc from the centromere. The distributions do not show a clear enrichment of essential genes over non-essential genes in pericentromeric regions. (TIF) [file pone.0312437.s002.tif]

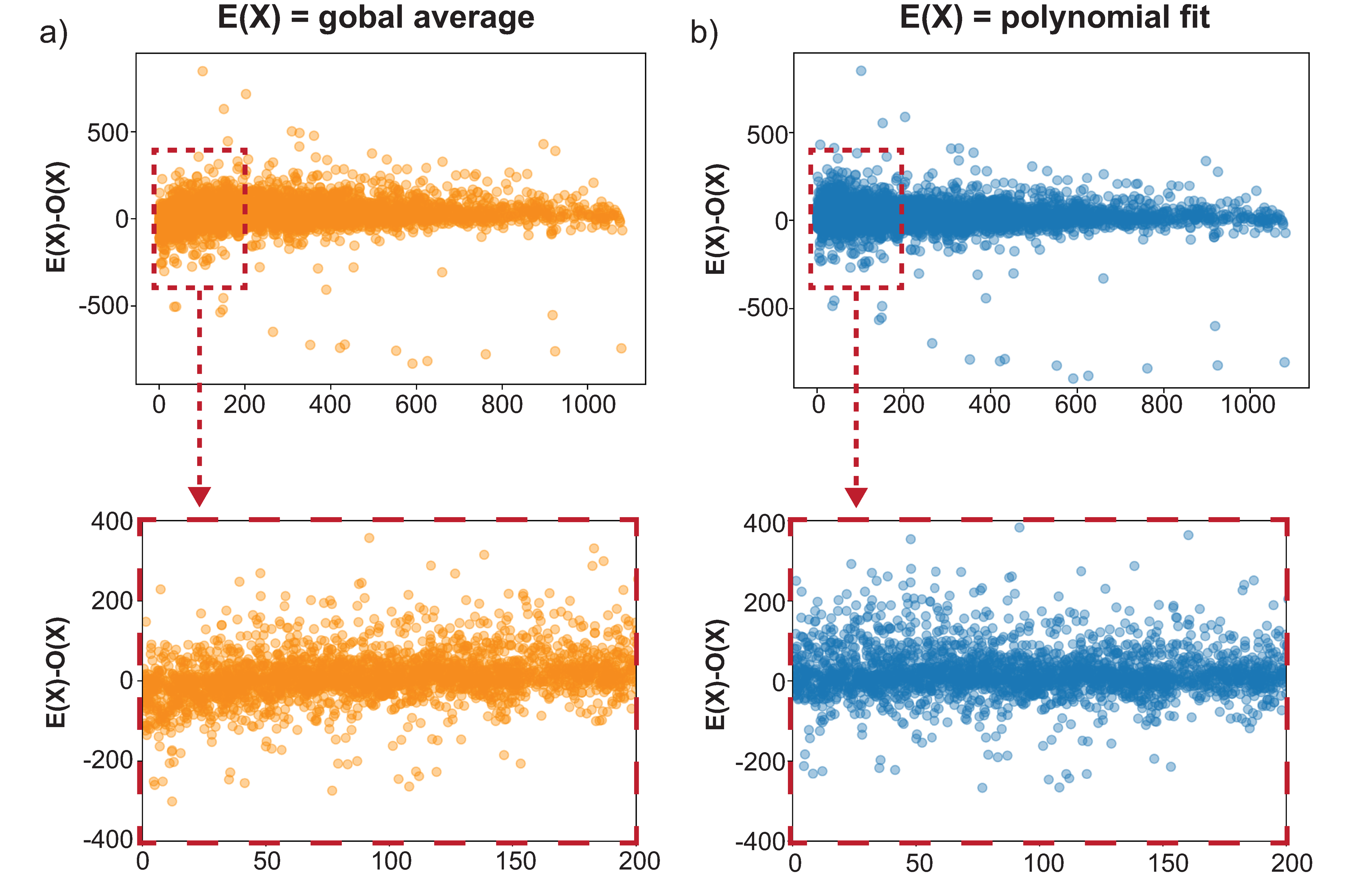

Supplement: S2 Fig — The difference between the expected (E(X)) and observed (O(X)) insertion density for all genes is plotted against the distance of the gene to the centromere. a) Using the global average to estimate the expected insertion rate results in a systematic overestimation of the insertion density of genes close to the centromere. This overestimation is visible as a skew towards negative values for genes close to the centromere. b) Using a polynomial fit of the observed insertion rate corrects for this skew. (TIF) [file pone.0312437.s003.tif]

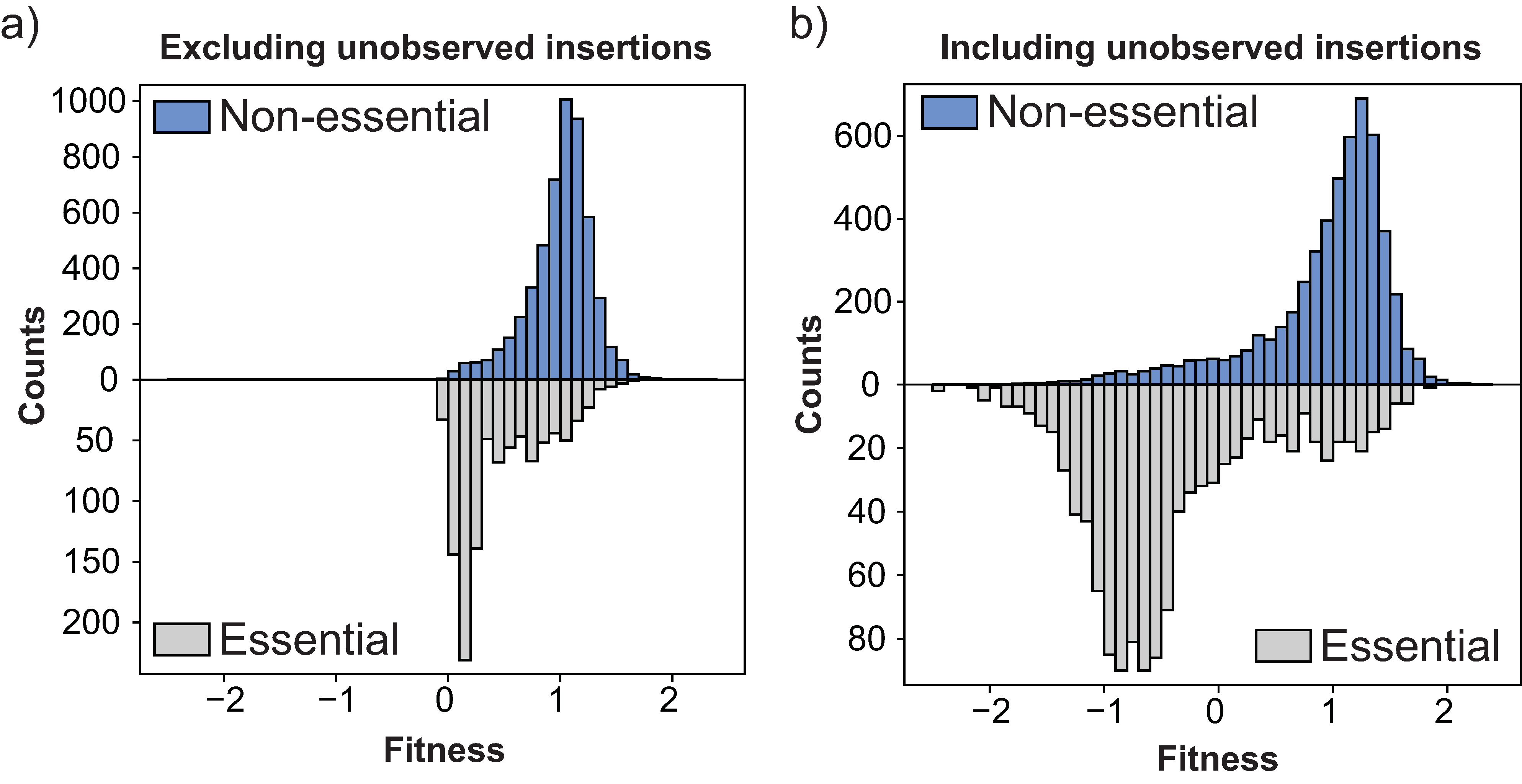

Supplement: S3 Fig — a) The fitness distribution of gene disruptions when unobserved insertions are excluded during the fitness estimation. In this case, the fitness estimates depend only on the average read count per insertion site. b) The fitness distribution of gene disruptions when unobserved insertions are excluded during the fitness estimation. The plot shows that the lower tail of the distribution has a larger spread when compared to (a), providing a better distinction between low-fitness genes. In this case, the fitness estimates of genes that have low fitness when disrupted depends on a combination of the average read count and the insertion density. (TIF) [file pone.0312437.s004.tif]
