# Supplementary material for: Saturated Transposon Analysis in Yeast as a one-step method to quantify the fitness effects of gene disruptions on a genome-wide scale
Source: PLoS One. 2025 Feb 6;20(2):e0312437. doi: 10.1371/journal.pone.0312437 (PMC11801604; doi:10.1371/journal.pone.0312437)
Supplement: S1 Table — (PDF) [file pone.0312437.s005.pdf]

Name	Purpose	Parent	Genotype	Reference
yLIC135	Parental strain	yLL3a	*Matα*<br>*can1-100*<br>*leu2-3,112*<br>*his3-11,15*<br>*ura3* ^{0}<br>*BUD4 from S288C*<br>*ade2Δ::*	[1]
yLIC137	Identification of genetic interactions of *BEM3* (data Fig. 7b)	yLIC135	*Matα*<br>*can1-100*<br>*leu2-3,112*<br>*his3-11,15*<br>*ura3* ^{0}<br>*BUD4 from S288C*<br>*ade2Δ::*<br>*bem3::natMX4*	This study
yWT01a	Identification of the genetic interactions of *BEM3* (data Fig. 7b)<br><br>Comparison with fitness values obtained from Bar-seq (data Fig. 7a)	yLIC135	*Matα*<br>*can1-100*<br>*leu2-3,112*<br>*his3-11,15*<br>*ura3* ^{0}<br>*BUD4 from S288C*<br>*ade2Δ::*	This study
