# Supplementary material for: Saturated Transposon Analysis in Yeast as a one-step method to quantify the fitness effects of gene disruptions on a genome-wide scale
Source: PLoS One. 2025 Feb 6;20(2):e0312437. doi: 10.1371/journal.pone.0312437 (PMC11801604; doi:10.1371/journal.pone.0312437)
Supplement: S2 Table — YNB: Yeast Nitrogen Base. CSM: Complete Supplement Mixture. Ura: Uracil. Ade: Adenine. (PDF) [file pone.0312437.s006.pdf]

**Step**	**Media**	**Components**
Preculture	SD-Ura+0.2% Glucose +2% Raffinose	• YNB w/o Amino Acids (6.8 g/L)<br>• CSM -Ura (0.77 g/L)<br>• Glucose (2 g/L)<br>• Raffinose (20 g/L)<br>• Adenine (20 mg/L)
Induction	SD-Ura+2% Galactose	• YNB w/o Amino Acids (6.8 g/L)<br>• CSM -Ura (0.77 g/L)<br>• Galactose (20 g/L)<br>• Adenine (20 mg/L)
Reseed	SD-Ade+2% Glucose	• YNB w/o Amino Acids (6.8 g/L)<br>• CSM -Ade (0.78 g/L)<br>• Glucose (20 g/L)
