# Supplementary material for: Saturated Transposon Analysis in Yeast as a one-step method to quantify the fitness effects of gene disruptions on a genome-wide scale
Source: PLoS One. 2025 Feb 6;20(2):e0312437. doi: 10.1371/journal.pone.0312437 (PMC11801604; doi:10.1371/journal.pone.0312437)
Supplement: S3 Table — (PDF) [file pone.0312437.s007.pdf]

#	Name	Barcode	Sequence (5'-3')
1	HT50_688_minidsSEQ1210	HT50	GCC ACA TAT TTA CCG ACC GTT ACC GAC CGT TTT CAT CCC TA
2	E2_HT48_MiniDS_RV	HT48	AGG TCA GTC ACA TGG TTA GGA CGC AGA GCT GAA ACG AAA ACG AAC GGG ATA AA
3	HT60_688_minidsSEQ1210	HT60	TAG GAT GAT TTA CCG ACC GTT ACC GAC CGT TTT CAT CCC TA
4	E2_HT49_MiniDS_RV	HT49	AGG TCA GTC ACA TGG TTA GGA CGC AGA TAG ACA ACG AAA ACG AAC GGG ATA AA
