# Supplementary material for: Saturated Transposon Analysis in Yeast as a one-step method to quantify the fitness effects of gene disruptions on a genome-wide scale
Source: PLoS One. 2025 Feb 6;20(2):e0312437. doi: 10.1371/journal.pone.0312437 (PMC11801604; doi:10.1371/journal.pone.0312437)
Supplement: S4 Table — (PDF) [file pone.0312437.s008.pdf]

| Step | Temperature | Time |
|---|---|---|
| 1 | 95 ºC | 3 min |
| | | |
| 2 | 95 ºC | 30 sec |
| 3 | 55 ºC | 30 sec |
| 4 | 72 ºC | 3 min |
| Go to step 2 (35x) | | |
| 6 | 72 ºC | 10 min |
| 7 | 4 ºC | Inf |
