# Supplementary material for: Saturated Transposon Analysis in Yeast as a one-step method to quantify the fitness effects of gene disruptions on a genome-wide scale
Source: PLoS One. 2025 Feb 6;20(2):e0312437. doi: 10.1371/journal.pone.0312437 (PMC11801604; doi:10.1371/journal.pone.0312437)
Supplement: S5 Table — Reads that map to the coding sequence of ADE2 were excluded when determining the reported values. (PDF) [file pone.0312437.s009.pdf]

Strain	Biological Replicates	# of clones	Technical Replicates	# of reads mapped	# of transposons mapped
yWT01a	B1	$7.1x10^6$	B1_T1	15341620	964082
			B1_T2	14133484	942484
			B1_T3	15803098	948445
			B1_T4	16963682	955841
			B1_T5	21601243	1012544
			B1_T6	18749208	980674
	B2	$8.6x10^6$	B2_T1	21301610	1107947
			B2_T2	17979884	1062241
			B2_T3	22407804	1152751
	B3	$7.9x10^6$	—	21870779	1010354
	B4	$9.9x10^6$	—	18792166	1168784
yLIC136	dnrp1-1		dnrp1-1_a	28071592	465974
			dnrp1-1_b	30122040	462828
	dnrp1-2		dnrp1-2_a	24836737	509141
			dnrp1-2_b	26363780	509014
yLIC137	—		—	11341842	597351
